# 8,8-Diethyl-1,4,5,8-tetra­hydro­naphthalene-1,4,5-trione

**DOI:** 10.1107/S1600536809001755

**Published:** 2009-01-17

**Authors:** Andrés Vega, Oney Ramirez-Rodríguez, Maximiliano Martínez-Cifuentes, Andrés Ibañez, Ramiro Araya-Maturana

**Affiliations:** a Universidad Andres Bello, Departamento de Ciencias Químicas, Av Republica 275, Santiago, Chile; b Departamento de Química Orgánica, Facultad de Ciencias Químicas y Farmacéuticas, Universidad de Chile, Chile; c CIMAT, Universidad de Chile, Av. Blanco Encalada 2008, Santiago, Chile

## Abstract

The title mol­ecule, C_14_H_14_O_3_, contains two fused six-membered carbon rings with keto groups at positions 1, 4 and 5 and a *gem*-diethyl group at position 8. The mol­ecule is close to planar (maximum deviation = 0.044 Å), with one ethyl group at each side of the mol­ecular plane, with exception of the keto group at position 1 which is slightly deviated from the plane and disordered over two positions one on each side of it (occupancies 0.80/0.20). The packing of the mol­ecule shows weak bonded chains along *a* through C—H⋯O contacts and two intramolecular C—H⋯O interactions are also present.

## Related literature

For the biologically active dimethyl analog, see: Araya-Maturana *et al*. (2002 ▶); for its use as a substrate for Diels-Alder cyclo­additions with 2,4-hexa­dienol, see: Araya-Maturana *et al*. (1999 ▶) and for the synthesis of biologically active compounds, see: Araya-Maturana *et al*. (2006 ▶); Mendoza *et al.* (2005 ▶); Rodríguez *et al.* (2007 ▶). For details of the synthesis of the 4,4-dimethyl analog, see: Castro *et al.* (1983 ▶); Vega *et al.* (2008 ▶).
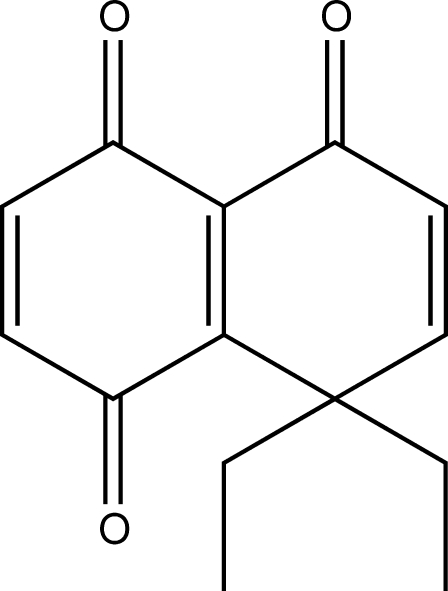


## Experimental

### 

#### Crystal data

C_14_H_14_O_3_
*M* *_r_* = 230.25
Orthorhombic, 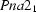

*a* = 12.7454 (8) Å
*b* = 10.8015 (7) Å
*c* = 8.8598 (5) Å
*V* = 1219.72 (13) Å^3^
*Z* = 4
Mo *K*α radiation
μ = 0.09 mm^−1^
*T* = 150 (2) K
0.49 × 0.48 × 0.46 mm

#### Data collection

Siemens SMART CCD area-detector diffractometer
Absorption correction: multi-scan (*SADABS*; Bruker, 1999 ▶) *T*_min_ = 0.958, *T*_max_ = 0.961
6581 measured reflections
2159 independent reflections
2119 reflections with *I* > 2σ(*I*)
*R*_int_ = 0.012

#### Refinement

*R*[*F* ^2^ > 2σ(*F* ^2^)] = 0.036
*wR*(*F* ^2^) = 0.099
*S* = 1.00
2159 reflections
166 parameters
15 restraints
H-atom parameters constrained
Δρ_max_ = 0.26 e Å^−3^
Δρ_min_ = −0.15 e Å^−3^

### 

Data collection: *SMART-NT* (Bruker, 2001 ▶); cell refinement: *SAINT-NT* (Bruker, 1999 ▶); data reduction: *SAINT-NT*; program(s) used to solve structure: *SHELXTL-NT* (Sheldrick, 2008 ▶); program(s) used to refine structure: *SHELXTL-NT*; molecular graphics: *SHELXTL-NT*; software used to prepare material for publication: *SHELXTL-NT*.

## Supplementary Material

Crystal structure: contains datablocks I, global. DOI: 10.1107/S1600536809001755/gw2058sup1.cif

Structure factors: contains datablocks I. DOI: 10.1107/S1600536809001755/gw2058Isup2.hkl

Additional supplementary materials:  crystallographic information; 3D view; checkCIF report

## Figures and Tables

**Table 1:** Hydrogen-bond geometry (Å, °)

*D*—H⋯*A*	*D*—H	H⋯*A*	*D*⋯*A*	*D*—H⋯*A*
C3—H3⋯O1^i^	0.95	2.27	3.207 (2)	169
C9—H9*A*⋯O2	0.99	2.40	3.014 (2)	120
C11—H11*B*⋯O2	0.99	2.39	3.027 (2)	122

Symmetry code: (i) 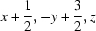.

**Fig. 1.**
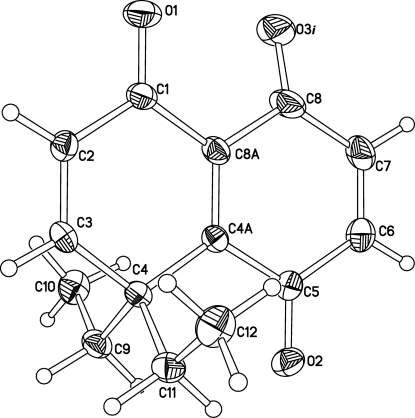
Molecular structure diagramas for I showing numbering scheme. Displacement ellipsoids are at 50% probability level and H atoms are shown as spheres of arbitrary radii. The less occupied disordered position (see experimental) for O3 (ii) was omitted for clarity.

**Fig. 2.**
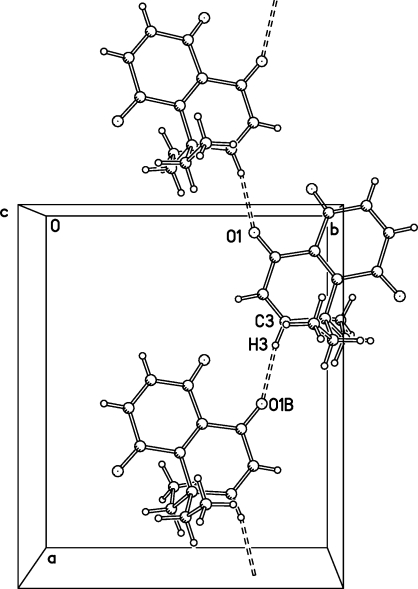
Packing structure of I showing weak bonded chains along a.

**Fig. 3.**
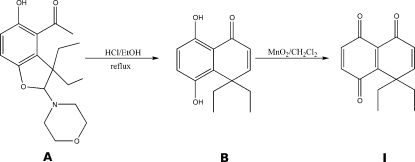
Preparation of the title compound.

## supplementary crystallographic information

### Comment

The title quinone (I) is closely related to its biologically active dimethyl analog 8,8-dimethylnaphtalene-1,4,5(8H)-trione (Araya-Maturana *et al.* 2002), which has been used as substrate for highly regioselective Diels-Alder cycloadditions with 2,4-hexadienol (Araya-Maturana *et al.* 1999) and for the synthesis of biologically active compounds (Rodríguez *et al.* 2007; Araya-Maturana *et al.* 2006; Mendoza *et al.* 2005). The ^1^H-NMR spectrum of the title compound exhibits equivalence of both ethyl groups, evidencing the existence of a symmetry plane in the molecule. It displays a single triplet for both methyl groups but two sextuplets for the methylene protons of the ethyl substituents; with couplings constant of 7.3 Hz and 15.4 Hz for the vecinal and geminal ones respectively. This evidences a rotational restriction for the chains. The non-equivalence of the signals of methylene protons in a non-chiral molecule could be envisoned supposing a rotational constrain exerted by the non bonding electrons of the near carbonyl group, avoiding the rotation of the bond between methylene groups and the quaternary carbon bearing the geminal ethyl groups. This hypotesis based on NMR solution data was tested for the present crystal structure.

The molecule I contains two six membered carbon rings fused, a *p*-quinone and a dienone core (Scheme 1). The dienone ring is highly planar mainly because of the insaturations in the carbon skeleton, while the quinonic framework displays a slightly distorted boat conformation, with one keto oxygen atom slightly out of the plane of the rest of the ring (see torsion angles). As described in the experimental section, the keto oxygen atom O3 is disordered over two positions of ocuppancy 0.80 and 0.20. placed at opposite sides of the molecular plane. This could be related to the equivalence of the methylene ^1^H-NMR signals in solution in the following way: the two conformations are probably very close (if not equal) in energy and rapid interconversion occurs in the NMR timescale. The situation is consistent with the observation of two positions for the keto oxygen atom in the crystal strcuture.

The crystal packing of the molecule shows weak bonded zigzag chains along the a cell axis, through C—H···O interactions, as depicted in Figure 2.

### Experimental

Synthesis of I. The title compound was prepared by oxydation of the corresponding hydroquinone B; obtained by rearrangement of the furane parent compound A (Vega *et al.*, 2008); with MnO_2_ as shown in Fig. 3. This procedure have been previously described for the 4,4-dimethyl analog (Castro *et al.*, 1983). X-ray quality crystals were obtained through recrystallization from benzene.

Spectroscopic Details. ^1^H and ^13^C NMR spectra were acquired using a Bruker AVANCE DRX 300 spectrometer operating at 300.13 MHz (^1^H) or 75.47 MHz (^13^C). All measurements were carried out at a probe temperature of 300 K. ^1^HNMR (CDCl_3_): 0.62(6*H*, t, *J* = 7.5 Hz, 2X CH_3_); 1.70(2*H*, dq, *J*_1_ = 7.5 Hz, *J*_2_ = 13.8 Hz, 2X CHH,); 2.52(2*H*, dq, *J*_1_ = 7.5 Hz, *J*_2_ = 13.8 Hz, 2X CHH); 6.53(1*H*, d, *J* = 10.2 Hz); 6.58(1*H*, d, *J* = 10.2 HZ). ^13^CNMR(CDCl_3_): 8.42, 31.41, 48.13, 130.77, 132.79, 135.32, 135.57, 152.59, 154.51, 182.46, 183.13, 186.76.

### Refinement

The hydrogen atoms positions were calculated after each cycle of refinement with *SHELXL* (Bruker,1999) using a riding model for each structure, with C—H distances in the range 0.96 to 1.00 Å. *U*_iso_(H) values were set equal to 1.5*U*_eq_ of the parent carbon atom for methyl groups and 1.2*U*_eq_ for the others. During the final stages of refinement some disorder on the position of the oxo oxygen atom O3 was evident. It was modelled using two positions labelled *i* and *ii* with partial occupation of 0.80 and 0.20 respectively.

### Crystal data

**Table:**

C_14_H_14_O_3_	*F*(000) = 488
*M**_r_* = 230.25	*D*_x_ = 1.254 Mg m^−^^3^
Orthorhombic, *P**n**a*2_1_	Mo *K*α radiation, λ = 0.71073 Å
Hall symbol: P 2c -2n	Cell parameters from 4760 reflections
*a* = 12.7454 (8) Å	θ = 24.9–50.1°
*b* = 10.8015 (7) Å	µ = 0.09 mm^−^^1^
*c* = 8.8598 (5) Å	*T* = 150 K
*V* = 1219.72 (13) Å^3^	Block, red
*Z* = 4	0.49 × 0.48 × 0.46 mm

### Data collection

**Table:**

Siemens SMART CCD area-detector diffractometer	2159 independent reflections
Radiation source: fine-focus sealed tube	2119 reflections with *I* > 2σ(*I*)
graphite	*R*_int_ = 0.012
φ and ω scans	θ_max_ = 25.1°, θ_min_ = 2.5°
Absorption correction: multi-scan (*SADABS*; Bruker, 1999)	*h* = −15→15
*T*_min_ = 0.958, *T*_max_ = 0.961	*k* = −12→12
6581 measured reflections	*l* = −10→10

### Refinement

**Table:**

Refinement on *F*^2^	Primary atom site location: structure-invariant direct methods
Least-squares matrix: full	Secondary atom site location: difference Fourier map
*R*[*F*^2^ > 2σ(*F*^2^)] = 0.036	Hydrogen site location: inferred from neighbouring sites
*wR*(*F*^2^) = 0.099	H-atom parameters constrained
*S* = 1.00	*w* = 1/[σ^2^(*F*_o_^2^) + (0.0642*P*)^2^ + 0.3077*P*] where *P* = (*F*_o_^2^ + 2*F*_c_^2^)/3
2159 reflections	(Δ/σ)_max_ < 0.001
166 parameters	Δρ_max_ = 0.26 e Å^−^^3^
15 restraints	Δρ_min_ = −0.15 e Å^−^^3^

### Special details

**Table:**

Experimental. 0.3 ° between frames and 10 secs exposure (per frame)
Geometry. All e.s.d.'s (except the e.s.d. in the dihedral angle between two l.s. planes) are estimated using the full covariance matrix. The cell e.s.d.'s are taken into account individually in the estimation of e.s.d.'s in distances, angles and torsion angles; correlations between e.s.d.'s in cell parameters are only used when they are defined by crystal symmetry. An approximate (isotropic) treatment of cell e.s.d.'s is used for estimating e.s.d.'s involving l.s. planes.
Refinement. Refinement of *F*^2^ against ALL reflections. The weighted *R*-factor *wR* and goodness of fit *S* are based on *F*^2^, conventional *R*-factors *R* are based on *F*, with *F* set to zero for negative *F*^2^. The threshold expression of *F*^2^ > σ(*F*^2^) is used only for calculating *R*-factors(gt) *etc*. and is not relevant to the choice of reflections for refinement. *R*-factors based on *F*^2^ are statistically about twice as large as those based on *F*, and *R*- factors based on ALL data will be even larger.

### Fractional atomic coordinates and isotropic or equivalent isotropic displacement parameters (Å^2^)

**Table:**

	*x*	*y*	*z*	*U*_iso_*/*U*_eq_	Occ. (<1)
O1	0.05513 (10)	0.73723 (13)	0.2345 (2)	0.0588 (5)	
C1	0.12734 (12)	0.80829 (16)	0.2581 (2)	0.0312 (4)	
C2	0.23531 (13)	0.76647 (15)	0.24985 (19)	0.0298 (4)	
H2	0.2495	0.6828	0.2240	0.036*	
C3	0.31458 (12)	0.84252 (16)	0.2775 (2)	0.0298 (4)	
H3	0.3834	0.8091	0.2713	0.036*	
C4	0.30503 (12)	0.97639 (16)	0.3177 (2)	0.0264 (4)	
C9	0.36538 (13)	1.04995 (17)	0.1926 (2)	0.0343 (4)	
H9A	0.3713	1.1375	0.2244	0.041*	
H9B	0.4373	1.0161	0.1842	0.041*	
C10	0.31363 (17)	1.04521 (19)	0.0381 (2)	0.0428 (5)	
H10A	0.3037	0.9587	0.0080	0.064*	
H10B	0.3585	1.0871	−0.0358	0.064*	
H10C	0.2454	1.0868	0.0426	0.064*	
C11	0.36382 (13)	0.99525 (17)	0.4703 (2)	0.0341 (4)	
H11A	0.4375	0.9680	0.4579	0.041*	
H11B	0.3648	1.0847	0.4944	0.041*	
C12	0.31612 (17)	0.92627 (19)	0.6018 (2)	0.0446 (5)	
H12A	0.2431	0.9523	0.6151	0.067*	
H12B	0.3559	0.9446	0.6938	0.067*	
H12C	0.3185	0.8371	0.5818	0.067*	
C4A	0.19164 (12)	1.01751 (15)	0.32711 (18)	0.0257 (3)	
C5	0.17023 (12)	1.15035 (15)	0.3703 (2)	0.0302 (4)	
O2	0.24055 (10)	1.22495 (10)	0.38734 (17)	0.0391 (3)	
C6	0.05978 (14)	1.18870 (18)	0.3909 (3)	0.0440 (5)	
H6	0.0453	1.2693	0.4283	0.053*	
C7	−0.01884 (14)	1.11471 (18)	0.3592 (3)	0.0444 (5)	
H7	−0.0887	1.1421	0.3759	0.053*	
C8	−0.00040 (15)	0.9904 (2)	0.2982 (3)	0.0515 (6)	
O3i	−0.07253 (14)	0.93529 (18)	0.2268 (3)	0.0604 (6)	0.80
O3ii	−0.0669 (5)	0.9189 (6)	0.3592 (11)	0.063 (2)	0.20
C8A	0.11022 (12)	0.94198 (15)	0.2963 (2)	0.0304 (4)	

### Atomic displacement parameters (Å^2^)

**Table:**

	*U*^11^	*U*^22^	*U*^33^	*U*^12^	*U*^13^	*U*^23^
O1	0.0288 (7)	0.0394 (7)	0.1082 (15)	−0.0062 (6)	−0.0007 (8)	−0.0163 (8)
C1	0.0240 (8)	0.0302 (8)	0.0396 (10)	−0.0027 (7)	0.0001 (7)	0.0002 (7)
C2	0.0308 (8)	0.0257 (8)	0.0329 (10)	0.0031 (6)	0.0016 (7)	−0.0017 (7)
C3	0.0214 (7)	0.0342 (8)	0.0338 (9)	0.0049 (6)	0.0014 (7)	0.0024 (7)
C4	0.0215 (7)	0.0294 (8)	0.0284 (8)	−0.0011 (6)	0.0010 (6)	0.0005 (7)
C9	0.0282 (8)	0.0360 (9)	0.0388 (10)	−0.0042 (7)	0.0057 (7)	0.0027 (7)
C10	0.0515 (12)	0.0436 (11)	0.0332 (10)	−0.0007 (9)	0.0069 (9)	0.0041 (8)
C11	0.0287 (8)	0.0394 (9)	0.0341 (9)	0.0005 (7)	−0.0061 (7)	−0.0012 (8)
C12	0.0541 (12)	0.0486 (11)	0.0311 (10)	0.0022 (9)	−0.0036 (9)	0.0016 (9)
C4A	0.0235 (7)	0.0284 (8)	0.0251 (8)	0.0014 (6)	−0.0006 (6)	0.0021 (6)
C5	0.0325 (8)	0.0293 (8)	0.0288 (8)	0.0035 (7)	−0.0009 (7)	0.0004 (7)
O2	0.0424 (7)	0.0303 (6)	0.0446 (8)	−0.0047 (5)	−0.0031 (6)	−0.0033 (6)
C6	0.0392 (10)	0.0339 (9)	0.0589 (13)	0.0110 (8)	0.0016 (10)	−0.0084 (10)
C7	0.0287 (9)	0.0454 (11)	0.0591 (12)	0.0122 (8)	0.0028 (9)	−0.0022 (9)
C8	0.0220 (8)	0.0379 (9)	0.0946 (17)	0.0012 (7)	0.0008 (10)	−0.0040 (11)
O3i	0.0286 (9)	0.0545 (11)	0.0980 (17)	0.0031 (8)	−0.0155 (11)	−0.0103 (12)
O3ii	0.016 (3)	0.050 (4)	0.122 (7)	−0.006 (3)	0.012 (4)	−0.032 (5)
C8A	0.0219 (8)	0.0308 (8)	0.0385 (9)	0.0031 (6)	0.0008 (7)	0.0020 (7)

### Geometric parameters (Å, °)

**Table:**

O1—C1	1.217 (2)	C11—H11A	0.9900
C1—C2	1.450 (2)	C11—H11B	0.9900
C1—C8A	1.499 (2)	C12—H12A	0.9800
C2—C3	1.325 (2)	C12—H12B	0.9800
C2—H2	0.9500	C12—H12C	0.9800
C3—C4	1.494 (2)	C4A—C8A	1.348 (2)
C3—H3	0.9500	C4A—C5	1.510 (2)
C4—C4A	1.514 (2)	C5—O2	1.215 (2)
C4—C11	1.559 (2)	C5—C6	1.479 (2)
C4—C9	1.566 (2)	C6—C7	1.312 (3)
C9—C10	1.520 (3)	C6—H6	0.9500
C9—H9A	0.9900	C7—C8	1.467 (3)
C9—H9B	0.9900	C7—H7	0.9500
C10—H10A	0.9800	C8—O3i	1.264 (3)
C10—H10B	0.9800	C8—O3ii	1.267 (3)
C10—H10C	0.9800	C8—C8A	1.504 (2)
C11—C12	1.511 (3)		
			
O1—C1—C2	120.84 (16)	C12—C11—H11B	108.7
O1—C1—C8A	122.44 (15)	C4—C11—H11B	108.7
C2—C1—C8A	116.72 (14)	H11A—C11—H11B	107.6
C3—C2—C1	121.40 (15)	C11—C12—H12A	109.5
C3—C2—H2	119.3	C11—C12—H12B	109.5
C1—C2—H2	119.3	H12A—C12—H12B	109.5
C2—C3—C4	125.59 (15)	C11—C12—H12C	109.5
C2—C3—H3	117.2	H12A—C12—H12C	109.5
C4—C3—H3	117.2	H12B—C12—H12C	109.5
C3—C4—C4A	112.01 (13)	C8A—C4A—C5	119.17 (14)
C3—C4—C11	107.09 (15)	C8A—C4A—C4	123.10 (14)
C4A—C4—C11	111.88 (13)	C5—C4A—C4	117.72 (13)
C3—C4—C9	106.41 (14)	O2—C5—C6	120.09 (15)
C4A—C4—C9	111.04 (13)	O2—C5—C4A	121.91 (14)
C11—C4—C9	108.14 (13)	C6—C5—C4A	118.00 (14)
C10—C9—C4	114.02 (15)	C7—C6—C5	122.00 (16)
C10—C9—H9A	108.7	C7—C6—H6	119.0
C4—C9—H9A	108.7	C5—C6—H6	119.0
C10—C9—H9B	108.7	C6—C7—C8	120.96 (16)
C4—C9—H9B	108.7	C6—C7—H7	119.5
H9A—C9—H9B	107.6	C8—C7—H7	119.5
C9—C10—H10A	109.5	O3i—C8—O3ii	56.0 (4)
C9—C10—H10B	109.5	O3i—C8—C7	119.90 (18)
H10A—C10—H10B	109.5	O3ii—C8—C7	107.1 (4)
C9—C10—H10C	109.5	O3i—C8—C8A	120.9 (2)
H10A—C10—H10C	109.5	O3ii—C8—C8A	114.8 (4)
H10B—C10—H10C	109.5	C7—C8—C8A	118.20 (17)
C12—C11—C4	114.25 (14)	C4A—C8A—C1	121.11 (14)
C12—C11—H11A	108.7	C4A—C8A—C8	120.61 (15)
C4—C11—H11A	108.7	C1—C8A—C8	118.27 (15)
			
O1—C1—C2—C3	−178.98 (19)	C4—C4A—C5—C6	−175.48 (16)
C8A—C1—C2—C3	1.2 (2)	O2—C5—C6—C7	173.1 (2)
C1—C2—C3—C4	−0.7 (3)	C4A—C5—C6—C7	−6.5 (3)
C2—C3—C4—C4A	1.2 (3)	C5—C6—C7—C8	−1.3 (3)
C2—C3—C4—C11	124.22 (18)	C6—C7—C8—O3i	−158.7 (3)
C2—C3—C4—C9	−120.31 (19)	C6—C7—C8—O3ii	141.2 (5)
C3—C4—C9—C10	68.10 (19)	C6—C7—C8—C8A	9.7 (3)
C4A—C4—C9—C10	−54.0 (2)	C5—C4A—C8A—C1	−177.87 (16)
C11—C4—C9—C10	−177.13 (15)	C4—C4A—C8A—C1	3.4 (3)
C3—C4—C11—C12	−64.03 (18)	C5—C4A—C8A—C8	2.6 (3)
C4A—C4—C11—C12	59.1 (2)	C4—C4A—C8A—C8	−176.14 (17)
C9—C4—C11—C12	−178.34 (15)	O1—C1—C8A—C4A	177.64 (19)
C3—C4—C4A—C8A	−2.6 (2)	C2—C1—C8A—C4A	−2.6 (2)
C11—C4—C4A—C8A	−122.82 (18)	O1—C1—C8A—C8	−2.9 (3)
C9—C4—C4A—C8A	116.25 (18)	C2—C1—C8A—C8	176.95 (18)
C3—C4—C4A—C5	178.65 (16)	O3i—C8—C8A—C4A	157.9 (2)
C11—C4—C4A—C5	58.39 (19)	O3ii—C8—C8A—C4A	−138.3 (5)
C9—C4—C4A—C5	−62.53 (18)	C7—C8—C8A—C4A	−10.3 (3)
C8A—C4A—C5—O2	−173.95 (17)	O3i—C8—C8A—C1	−21.6 (3)
C4—C4A—C5—O2	4.9 (2)	O3ii—C8—C8A—C1	42.2 (5)
C8A—C4A—C5—C6	5.7 (3)	C7—C8—C8A—C1	170.2 (2)

### Hydrogen-bond geometry (Å, °)

**Table:**

*D*—H···*A*	*D*—H	H···*A*	*D*···*A*	*D*—H···*A*
C3—H3···O1^i^	0.95	2.27	3.207 (2)	169
C9—H9A···O2	0.99	2.40	3.014 (2)	120
C11—H11B···O2	0.99	2.39	3.027 (2)	122

Symmetry codes: (i) *x*+1/2, −*y*+3/2, *z*.
